# Do fiber tips with different geometric designs affect intracanal medicament removal in laser-activated irrigation of immature permanent teeth?: an in vitro study

**DOI:** 10.1007/s10266-026-01390-8

**Published:** 2026-04-13

**Authors:** Merve IŞIK AYDIN, Amine YİĞİT

**Affiliations:** Department of Endodontics, Faculty of Dentistry, Bolu Abant Izzet Baysal University, Bolu, Turkey

**Keywords:** Endodontics, Regenerative endodontics, Lasers, Calcium hydroxide, Metronidazole, Ciprofloxacin

## Abstract

This in vitro study aimed to evaluate the effects of fiber tips with different geometric designs on the removal of intracanal medicaments during laser-activated irrigation (LAI) in immature permanent teeth. A total of 110 mandibular premolars were prepared using standardized procedures to simulate immature permanent tooth models. The teeth were randomly assigned to two groups according to the intracanal medicament applied. Calcium hydroxide (CH) and double antibiotic paste (DAP) were removed using standard needle irrigation (SNI) and LAI protocols, namely PIPS and SWEEPS, with two different fiber-tip end designs: flat (F) and radial (R). After irrigation, the specimens were examined under a stereomicroscope and digital images were obtained. The efficacy of medicament removal was quantitatively assessed by calculating the percentage area of residual medicament within the canal space using image analysis software (ImageJ). A significant difference was observed between the CH and DAP groups in terms of residual medicament levels (p < 0.05), with lower residue values in the CH group. Significant differences were also found among the irrigation activation protocols (p < 0.05). SNI showed the lowest removal efficiency, whereas SWEEPS-R resulted in the lowest residual medicament values. Within the limitations of this in vitro study, the SWEEPS-R protocol showed the highest removal efficiency for both CH and DAP, whereas SNI resulted in the greatest amount of residual medicament. CH was removed more effectively than DAP, and none of the tested protocols achieved complete removal. Understanding the influence of fiber-tip geometry on medicament removal may help guide the selection of more effective LAI protocols for regenerative endodontic procedures in immature permanent teeth.

## Introduction

Immature permanent teeth with pulp necrosis caused by caries, trauma, or developmental disorders present significant clinical challenges because of their short roots, wide canals, thin dentin walls, and open apices, which limit the effectiveness of conventional root canal treatment [1, 2]. In these cases, regenerative endodontic treatment (RET) has gained prominence as a biologically based approach aimed at reestablishing the pulp-dentin complex [3]. According to the guidelines established by the American Association of Endodontists (AAE) and the European Society of Endodontology (ESE), RET encompasses a series of steps, including thorough canal disinfection, activation of stem cells and signaling molecules, and scaffold formation through induced bleeding to facilitate tissue regeneration [1, 4]. In RETs, achieving adequate disinfection of the root canal system remains challenging, as mechanical instrumentation is intentionally limited [5]. Therefore, the implementation of optimized irrigation strategies, adjunctive activation techniques, and effective intracanal medicaments is crucial for achieving adequate disinfection and ensuring successful regenerative outcomes [6, 7].

In RET, the primary intracanal medicaments used for canal disinfection include triple antibiotic paste (TAP), composed of metronidazole, ciprofloxacin, and minocycline; double antibiotic paste (DAP), containing metronidazole and ciprofloxacin; and calcium hydroxide (CH) [8]. Although these agents exhibit broad antimicrobial activity, their application has been associated with several adverse effects, such as the promotion of antibiotic-resistant microorganisms within biofilms, cytotoxicity toward stem cells, coronal discoloration, and reduced dentin structural integrity and fracture resistance [9, 10]. Therefore, the effective removal of these medicaments from the canal walls is considered important to minimize potential complications and support success outcomes in RET procedures [11].

Intracanal medicament removal is typically achieved using irrigation solutions combined with mechanical preparation [12]. However, in RET, the limited use of chemo-mechanical instrumentation may impede adequate medicament elimination, potentially compromising the treatment outcomes. Current AAE and ESE guidelines recommend the use of ethylene diamine tetraacetic acid (EDTA) in conjunction with standard needle irrigation (SNI) for this purpose [1, 4]. Despite the widespread clinical use of SNI, irrigation penetration remains limited due to factors such as the inability to insert the irrigation needle up to the working length and the design of the needle tip [13, 14]. This may negatively affect the effectiveness of three-dimensional disinfection of the root canal system, particularly compromising intracanal medicament removal in teeth with immature open apexes [14]. Therefore, alternative irrigation activation methods are recommended to enhance irrigation effectiveness and intracanal disinfection [15].

Laser-activated irrigation (LAI) enhances the efficacy of irrigants and improves root canal disinfection efficacy. Photon-induced photoacoustic streaming (PIPS; Fotona, Ljubljana, Slovenia), an Er:YAG laser technique, generates three-dimensional irrigant movement through cavitation and photoacoustic shock waves, significantly increasing debridement and antimicrobial effectiveness [16]. Recently, shock wave-enhanced emission photoacoustic streaming (SWEEPS; Fotona, Ljubljana, Slovenia) was developed to further improve the PIPS performance [17]. In SWEEPS, rapid sequential bubble formation and collapse intensify shock wave generation, enabling efficient irrigant activation even in narrow canal spaces and enhancing the penetration and disinfection capacity of irrigants [18]. The hydrodynamic effects produced during LAI may vary depending on the fiber-tip geometric design. Flat tips predominantly emit energy forward, whereas radial tips distribute emission laterally, which may lead to different fluid dynamics and cavitation patterns during activation [19].

The effectiveness of LAI methods in removing intracanal medicaments has been demonstrated in several studies [20, 21]. However, no study has been conducted on the intracanal medicament removal efficacy of LAI applied using fiber tips with different geometric designs in simulated immature permanent tooth models. Therefore, the influence of fiber-tip geometry on intracanal medicament removal efficiency in immature tooth models remains unclear. This study aimed to evaluate the effectiveness of LAI using fiber tips with different geometric designs in removing intracanal medicaments from simulated immature permanent teeth. The null hypothesis of this study was that there would be no difference in the medicament removal effectiveness of irrigation activation applied with LAI tips of different geometric designs.

## Materials and methods

**Fig. 1:**
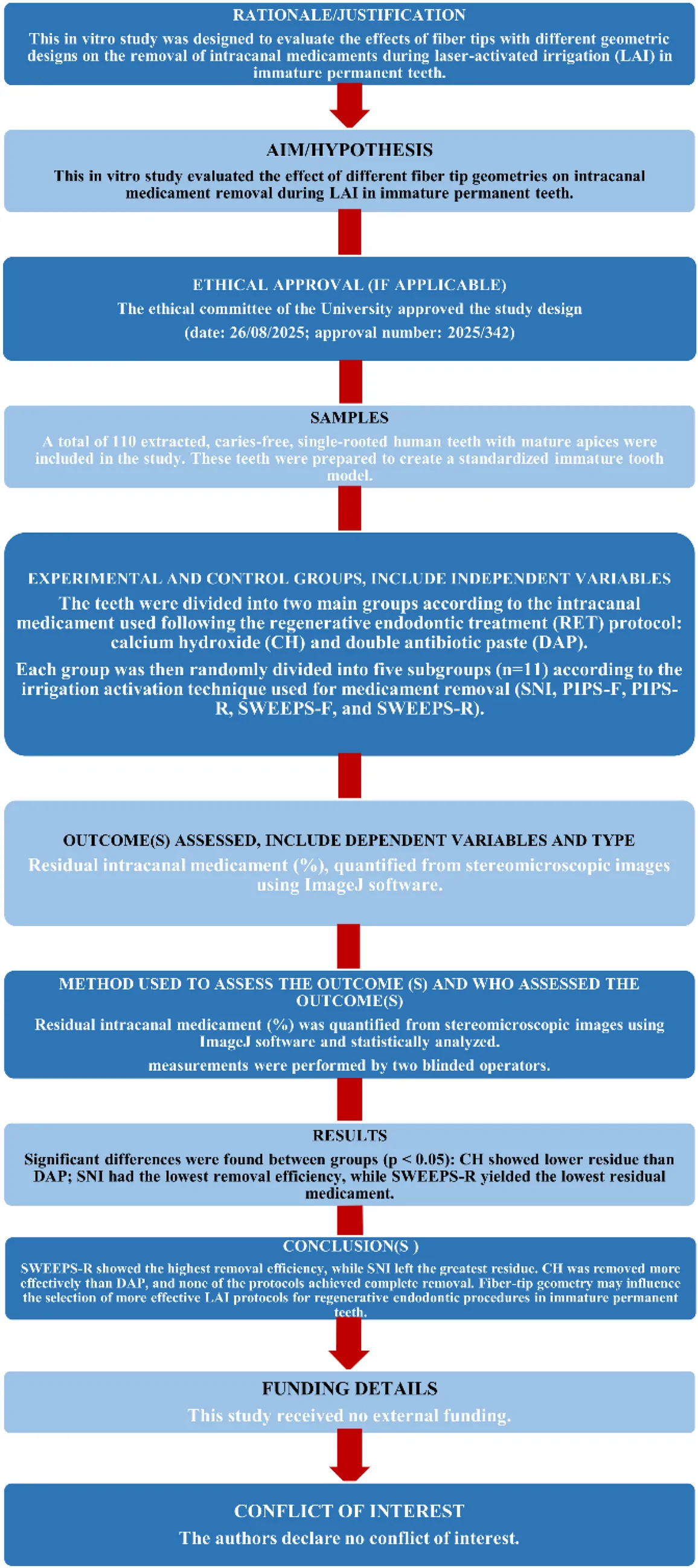
Flowchart of the study design according to PRILE guidelines

The manuscript of this laboratory study has been written according to the Preferred Reporting Items for Laboratory Studies in Endodontology (PRILE) 2021 guidelines [22] (Fig. 1).

### Ethics committee approval

This study was approved by the Bolu Abant İzzet Baysal University Non-Interventional Research Ethics Committee (date: 26/08/2025; approval number: 2025/342).

### Sample selection, preparation and placement of intracanal medicaments

Relevant studies were reviewed to guide the sample size calculation, and the highest required number derived from the statistical methods aligned with the primary hypotheses was selected. Using G*Power 3.1.9.2 (Heinrich-Heine-University Düsseldorf, Germany) [23], the standardized effect size was determined to be 0.5125 based on a comparable study [24] with a 95% confidence level (α = 0.05) and a theoretical power of 0.80, the minimum sample size required for each group was calculated to be 11. Carious-free, single-rooted human mandibular premolars with closed apex were collected. All teeth were examined under a stereomicroscope (Olympus SZ61, Tokyo, Japan) to exclude specimens with fractures or anatomical anomalies. After removing periodontal tissue remnants from the root surfaces of 110 eligible teeth using a periodontal curette, the samples were stored in a 0.1% thymol solution at 4 °C until use.

Immature tooth models were prepared following the methodology described in a previous study [25]. The apical 3 mm of each root was resected, and the root length was standardized to 14 ± 1 mm. Therefore, considering that an apical diameter of 1.1 mm is critical for RET, root canals were enlarged using Gates-Glidden drills (#2–#4; Proud, London, United Kingdom) until they reached a diameter of 1.1 ± 0.1 mm [26]**.** Saline irrigation with SNI was performed between each instrument. Subsequently, each canal was irrigated with 20 mL of 1.5% sodium hypochlorite (NaOCl) for 5 min, followed by 20 mL of saline and dried with paper points in accordance with the AAE and ESE recommendations [1, 4]. To simulate clinical conditions during irrigation, the apical root ends were sealed with modeling wax, and suction aspiration was applied.

The teeth were randomly allocated into two main groups according to the intracanal medicament used. All medicaments were prepared according to the manufacturer’s instructions and delivered into the canals using a Lentulo spiral (VDW, Munich, Germany). Before placing the intracanal medicament, the modeling wax was briefly removed from the root apex to ensure that the canal lumen was completely filled. The medicaments were prepared as follows:

DAP (*n* = 55): Equal proportions of metronidazole (Flagyl, Sanofi, Istanbul, Turkey) and ciprofloxacin (Cipro, Biofarma, Istanbul, Turkey) were mixed with distilled water to obtain a final concentration of 1–5 mg/mL.

CH (*n* = 55): Calcium hydroxide powder (Kalsin, Aktu Tic., Izmir, Turkey) was mixed with distilled water at a 1:1 ratio.

Following confirmation of adequate medicament placement, the modeling wax was repositioned, and the access cavities were sealed with a temporary restorative material (Cavitimi, Imicryl Dental, Turkey). All specimens were stored at 37 °C and 95% relative humidity for three weeks to simulate clinical RET procedures.

### Specimen allocation and randomization

All procedures were performed under standardized conditions by two operators (M.I.A. and A.Y.). After temporary sealing of the access cavities, specimens in the CH and DAP groups were randomly assigned to the irrigation activation subgroups using a simple randomization protocol. The allocation sequence was generated using a random number generator (Microsoft Excel, RAND function, Version 16.0) by an independent endodontist who was not involved in the experimental procedures or outcome assessment. Each specimen was assigned a unique numerical code, and all subsequent procedures were conducted using coded samples. Outcome assessment was performed on coded stereomicroscopic images, and the operators remained blinded to group allocation during image analysis.

### Removal of intracanal medicaments

After a 3-week incubation period, all teeth treated with DAP or CH were randomly assigned to five subgroups (n = 11) according to the irrigation activation method. Each specimen was irrigated with 20 mL of 17% EDTA, followed by saline, as recommended by the ESE guidelines [1]. EDTA enhances the release of dentin-derived growth factors that promote stem cell differentiation, whereas a final saline rinse is advised to minimize the potential adverse effects of irrigants on target cells [1]. To simulate the clinical irrigation dynamics, the apices were sealed with modeling wax throughout the irrigation and retained until sectioning. Activation protocols were performed strictly according to the manufacturer’s instructions, as described below.

#### SNI

A 30-gauge side-vented closed-end needle (Endo-Top; Cerkamed, Stalowa Wola, Poland) was positioned 1 mm short of the working length and moved vertically with gentle movements. This needle design was selected to minimize the risk of irrigant extrusion in the simulated immature open-apex model, in accordance with current RET clinical recommendations that advocate irrigation systems designed to reduce extrusion (e.g., a closed-end, side-vented needle) and positioning the needle approximately 1 mm short of the root end [4]. This approach is also consistent with the ESE position statement, which recommends the use of a side-vented needle during irrigation in RET procedures [1]. Each 5 mL of EDTA and saline was delivered for 20 s.

#### PIPS F

**Fig. 2:**
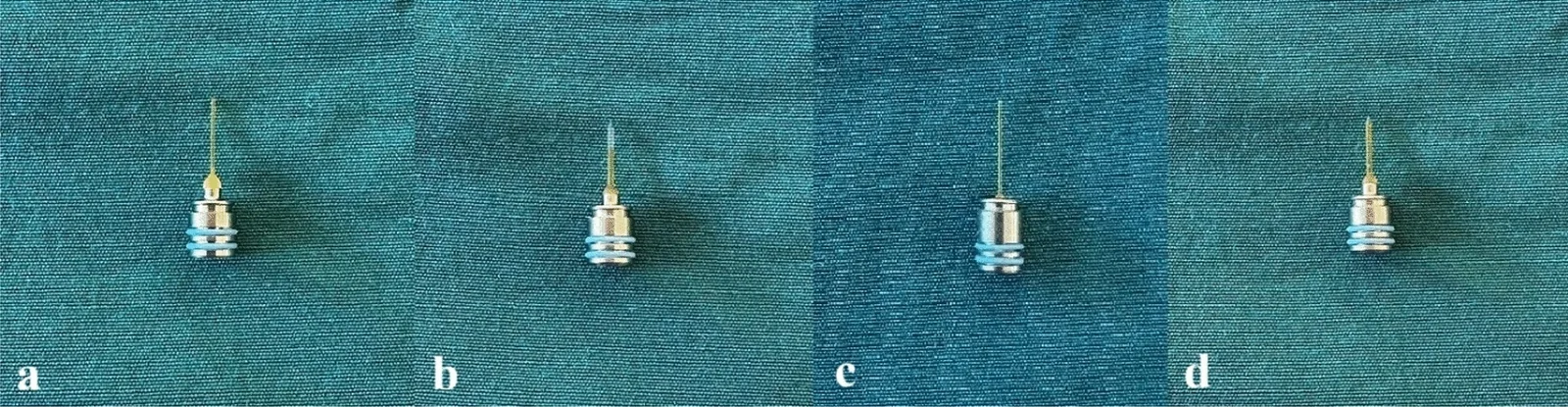
Laser fiber tips with different geometric designs used in the study (**a** PIPS F, **b** PIPS R, **c** SWEEPS F and **d** SWEEPS R)

The Fotona Light Walker Er:YAG laser (LightWalker AT; Fotona, Ljubljana, Slovenia) was set in Super Short Pulse (SSP) mode with water and air off, according to the manufacturer’s instructions, at 20 mJ, 15 Hz, 0.3 W. A PIPS (Fotona, Ljubljana) flat fiber tip (400/9 mm) (Fig. 2a) was placed in the reservoir area. Four activation cycles of 20 s each were performed using the solutions in the order and volume specified in the SNI. Each 5 mL of solution was activated for 20 s.

#### PIPS R

Using identical laser parameters, the PIPS radial fiber tip (600/9 mm) (Fig. 2b) was placed in the reservoir, and activation was performed in the same sequence as that for PIPS-F.

#### SWEEPS F

The SWEEPS protocol was applied using the LightWalker Er:YAG laser (Fotona) in automatic SWEEPS mode (20 mJ, 15 Hz, 0.3 W; water/air off). A flat SWEEPS fiber tip (300/9 mm) (Fig. 2c) was coronally placed, and four cycles of 20-s activation were performed for each solution.

#### SWEEPS R

Using the same laser settings, the SWEEPS radial fiber tip (600/9 mm) (Fig. 2d) was applied, and activation was completed according to the protocol described for SWEEPS-F.

### Evaluation of remaining intracanal medicaments

Following irrigation activation the modeling wax was removed, and each tooth was longitudinally sectioned using a diamond disc without invading the canal space of the tooth. Once an adequate groove was created, an enamel chisel was placed into the notch, and gentle pressure was applied to separate the two halves [27]. Each specimen was examined under a stereomicroscope (Olympus SZ61) at 20 × magnification, and images were captured using a digital camera (Olympus DP12; Olympus, Tokyo, Japan). Images were analyzed using ImageJ software (ImageJ 1.47 V; National Institutes of Health, USA). The outline of the root canal lumen was first delineated and designated as %100. The area containing residual intracanal medicament was measured, and the values were converted into percentage of remnants. This in vitro study employed a factorial design including two intracanal medicaments and five irrigation activation methods. The primary outcome was the percentage area of residual medicament quantified by image analysis. To improve interpretability and statistical sensitivity, analyses were performed using percentage data. The obtained percentage data were statistically analyzed. The reliability of repeated measurements was assessed using the intraclass correlation coefficient (ICC) based on the percentage values obtained from ImageJ analysis. The ICC value was 0.984, indicating excellent agreement between the measurements.

### Statistical analysis

The data were analyzed using the R program (v4.5.0; R Foundation for Statistical Computing, Vienna, Austria). The conformity of the data to a normal distribution was examined with the Shapiro–Wilk test. For the comparison of values that did not conform to the distribution according to group and activation method factors, ART Anova was used with the ARTool package, and multiple comparisons were made using Holm correction. The results of the analysis were presented as median (minimum: maximum) for quantitative data. The significance level was set at p < 0.05.

## Results

**Fig. 3:**
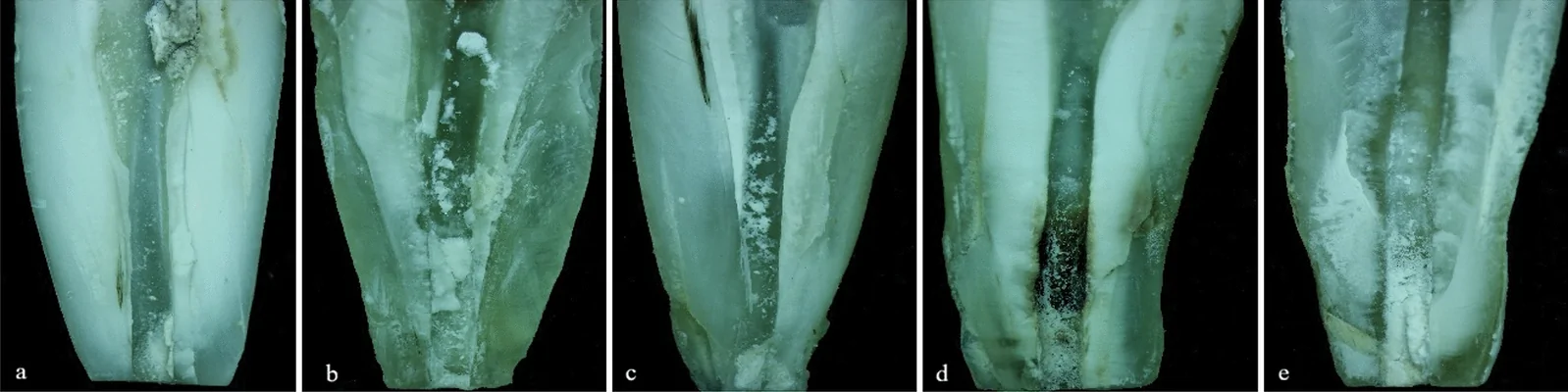
Representative stereomicroscopic images showing residual intracanal medicaments after irrigation activation in the CH group (**a** SNI, **b** PIPS F, **c** PIPS R, **d** SWEEPS F and **e** SWEEPS R)

**Fig. 4:**
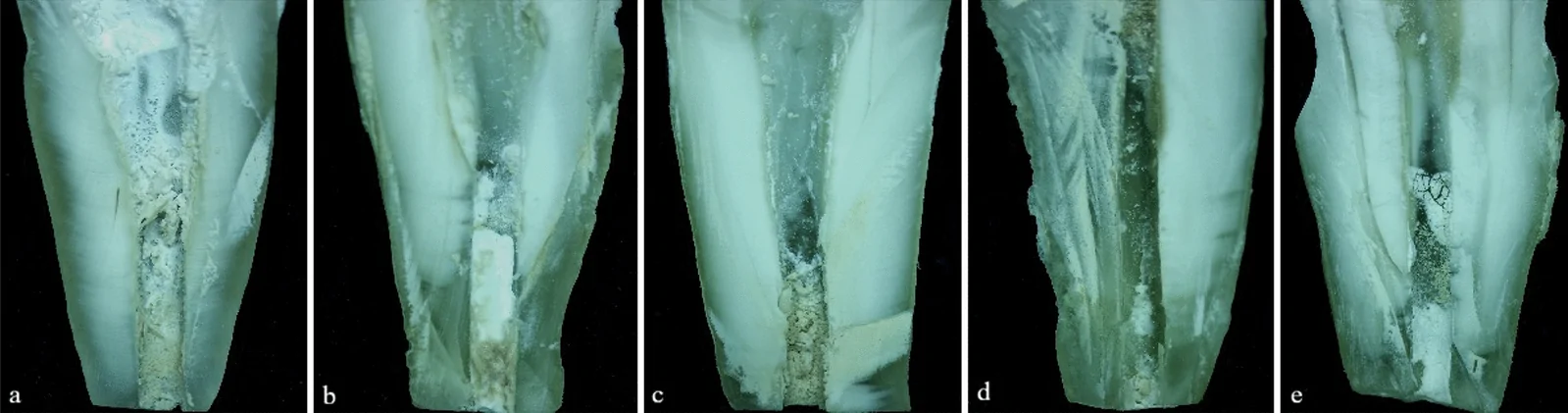
Representative stereomicroscopic images showing residual intracanal medicaments after irrigation activation in the DAP group (**a** SNI, **b** PIPS F, **c** PIPS R, **d** SWEEPS F and **e** SWEEPS R)

**Fig. 5:**
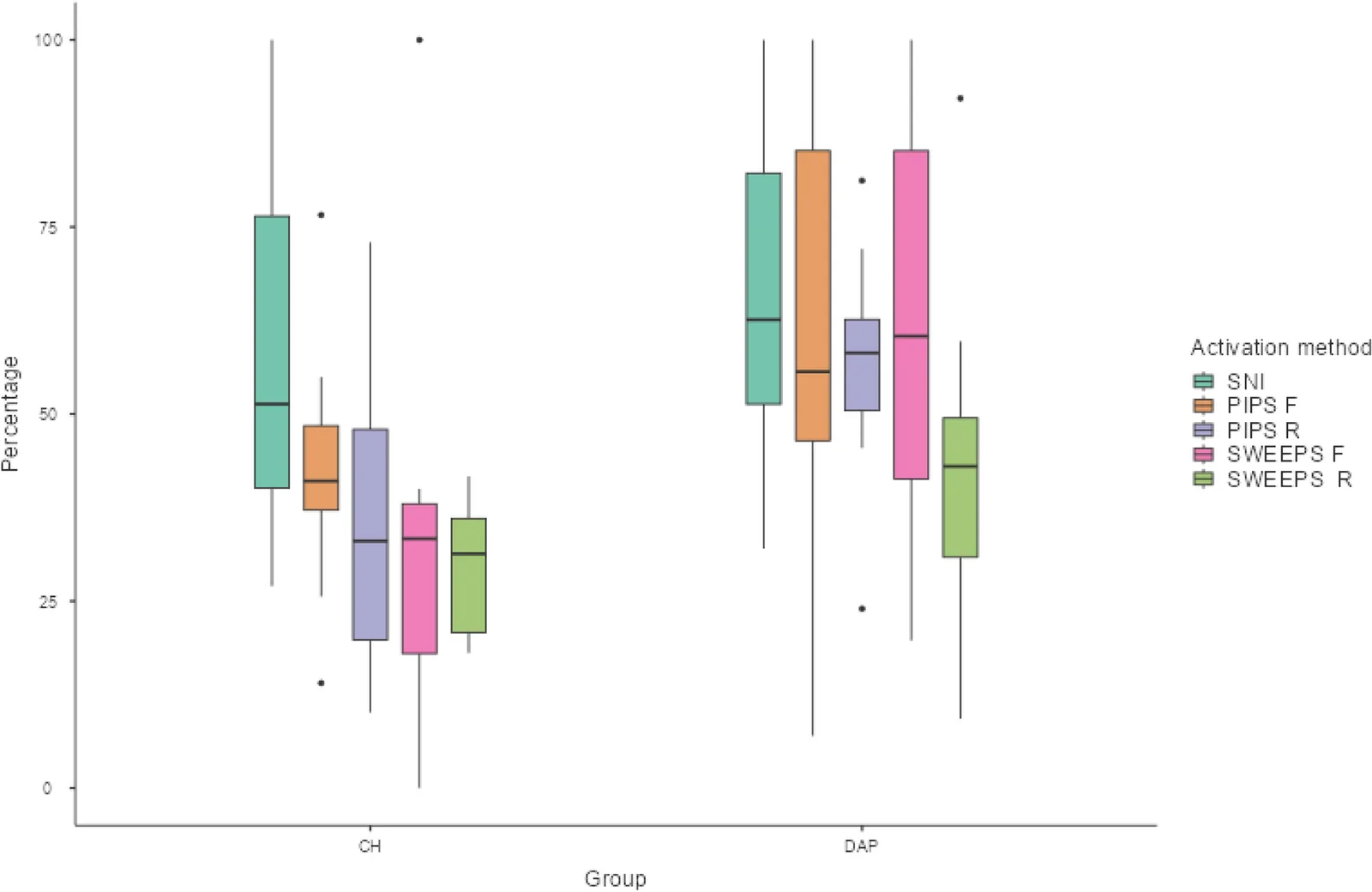
Box plot of the percentage variable by group and activation method factors

Regardless of the irrigation activation method, the median values of remaining intracanal medicament percentages showed statistically significant differences between the groups (p < 0.05). The median percentage of remaining medicament was found to be 37.288 in the CH group and 55.645 in the DAP group. This finding indicates that medicament removal from the root canal was greater in the CH group compared to the DAP group. The median values of remaining intracanal medicament percentages also differed significantly according to the irrigation activation methods (p < 0.05) (Figs. 3 and 4). The median values were determined as 57.186 for SNI, 48.414 for PIPS-F, 49.824 for PIPS-R, 38.595 for SWEEPS-F, and 34.206 for SWEEPS-R. In pairwise comparisons, a statistically significant difference was observed between SNI and SWEEPS-R, and between PIPS-F and SWEEPS-R (p < 0.05). Among the tested irrigation activation methods, the lowest median percentage of remaining intracanal medicament was observed in the SWEEPS-R group. The interaction between type of medicament and irrigation activation method was not found to be statistically significant with respect to the median values of remaining intracanal medicament percentage (p > 0.05) (Table 1, Fig. 5).

**Table 1:** Comparison of residual intracanal medicament percentages according to medicament type and irrigation activation method

Irrigation activation method	Group		Total		Test statistic	p^x^	ES
	CH	DAP					
SNI	51.327 (27: 100)	62.595 (32: 100)	57.186 (27: 100)^b^	Group	23.409	** < 0.001**	0.190
PIPS F	41.041 (14.04: 76.6)	55.645 (7: 100)	48.414 (7: 100)^b^	Irrigation activation method	4.870	**0.001**	0.163
PIPS R	33.028 (10.1: 72.97)	58.163 (23.97: 81.19)	49.824 (10,1: 81,19)^ab^	Group*Irrigation activation method	0.996	0.413	0.038
SWEEPS F	33.333 (0: 100)	60.396 (19.74: 100)	38.595 (0: 100)^ab^				
SWEEPS R	31.313 (18.06: 41.67)	43 (9.28: 92.19)	34.206 (9.28: 92.19)^a^				
Total	37.288 (0: 100)	55.645 (7: 100)					

Bold values represent statistically signifi cant results, where p-values are less than 0.05

^x^Art Anova; The median has been used as the comparison method. Median (minimum: maximum); ES: Effect size, Partial eta square (η^2^_p_); ^a−b^ There is no difference between activation methods with the same letter

## Discussion

RET aims to continue or reactivate the biological process of tooth development, relieve pulp necrosis and post-traumatic symptoms in immature permanent teeth, and maintain the function of the tooth in the mouth [28]. However, the complex and variable nature of the treatment process may affect the success of the treatment, depending on the changes made in the regeneration protocol [29]. The thin dentin walls of immature permanent teeth limit mechanical preparation; therefore, the use of intracanal medicaments is recommended to ensure effective canal disinfection [30, 31]. However, intracanal medicaments may influence the placement and sealing ability of coronal barrier materials, and their potential adverse effects on stem cells underscore the importance of promoting their removal from the root canal [32]. Consequently, the search for an irrigation activation technique that can safely and effectively remove intracanal medicaments from immature teeth remains ongoing. Therefore, the present study aimed to evaluate the effectiveness of LAI systems with different fiber tip geometries in removing intracanal medicaments from simulated immature permanent teeth, compared with SNI. To the best of our knowledge, this study is among the first to investigate the influence of fiber tip geometry on intracanal medicament removal in an immature tooth model. In this context, the study links the physical principles underlying LAI, particularly the hydrodynamic effects generated by PIPS and SWEEPS fiber tips, with a measurable medicament removal outcome. The observed differences in residual intracanal medicament among the tested irrigation activation techniques led to the rejection of the null hypothesis.

Ensuring standardization beyond the parameters tested in in vitro studies is crucial for the reliability of the results obtained. It has been reported that to ensure standardization in immature permanent teeth where the in vitro RET protocol is planned to be applied, samples with similar apical diameters and root canal sizes from the same tooth group should be used [11]. In this regard, mandibular premolars with similar root-crown ratios and anatomical features were preferred in the current study. To ensure dimensional standardization in the samples included in the study, standardization was achieved by removing a certain amount of tooth tissue from the crown and root apical regions [25], and then making apical diameter preparations, as reported by Hançerlioğulları et al. [22]. In addition, the type of irrigation solution, the volume used, the activation technique and its duration are stated in the literature as important variables affecting the results of endodontic treatment [33]. Therefore, the same irrigation and activation protocols were applied to all groups and samples tested in our study.

Studies evaluating the removal of intracanal medicaments have reported the use of various imaging methods, such as digital photographs [34], dental operating microscopes [35], stereomicroscopes [36], scanning electron microscopes [37], cone beam computed tomography [38], and micro-computed tomography [39]. However, it has been stated that two-dimensional examination techniques, such as dental operating microscopes, stereomicroscopes, and digital photography, can only evaluate the superficial layer remaining on the canal walls and cannot provide three-dimensional information about the removal depth, and volumetric analyses performed with micro-computed tomography can provide more accurate results [40, 41]. However, factors that may negatively affect image quality, such as the limited availability of micro-computed tomography, its high cost, long scanning and reconstruction time, and the different radiopacities of the intracanal medicaments used, make it difficult to apply this method in every study [42, 43]. Therefore, in this study, the quantitative evaluation of the amount of medicaments remaining in the root canal space was carried out by calculating the percentage of the remaining medicament area on standardized stereomicroscope images at 20 × magnification using the ImageJ program, as it also facilitates ease of application.

The physico-chemical properties of intracanal medicaments are considered among the important factors that may affect their removability from the root canal system. Due to its relatively small particle size, CH may disperse more easily along the root canal walls. In contrast, the diffusion characteristics of antibiotic pastes such as DAP may vary depending on their particle structure and the carrier used. These properties may contribute to the persistence of residual medicament after irrigation procedures. Previous studies have also suggested that the particle size and diffusion characteristics of intracanal medicaments may influence their removal from the root canal system [11, 44]. However, in the present study, residual medicament was evaluated based on two-dimensional measurements of the root canal surface; therefore, the possible penetration of medicaments into dentinal tubules was not directly assessed.

Previous studies investigating the removal of intracanal medicaments using irrigation activation methods have reported inconsistent results. Berkhoff et al. [11] reported that CH was removed more effectively than TAP regardless of the irrigation technique used and suggested that this finding might be related to the higher diffusion capacity of TAP. In contrast, Eymirli et al. [45] showed that, when LAI was applied, the amount of residual CH was significantly higher than that of TAP and attributed this finding to the smaller particle size of CH, which may facilitate its distribution within dentin. Usta et al. [46] reported that the removability of CH and DAP was similar in an immature permanent tooth model regardless of the irrigation activation method. In the present study, however, when the medicaments were evaluated separately, CH showed lower residual amounts than DAP. These discrepancies among studies may be attributed to differences in study design, technical variables such as laser tip design, power settings, irrigant volume and concentration, as well as the physico-chemical properties of the medicaments and the diversity of evaluation methods used. Although the effective removal of intracanal medicaments is generally considered important in RET procedures, it should also be recognized that these medicaments may exert beneficial biological effects. Previous studies have reported that certain intracanal medicaments and irrigation protocols can stimulate the release of growth factors, such as TGF-β1 and VEGF, from the dentin matrix [47, 48]. These bioactive molecules play a key role in stem cell migration, proliferation, and differentiation, thereby contributing to the regenerative process. Therefore, while the removal of intracanal medicaments is important to minimize possible interference with biomaterials or stem cell viability, their potential role in promoting the release of bioactive dentin matrix components should also be taken into consideration [47].

In the present study, the effectiveness of irrigation activation performed with PIPS and SWEEPS using fiber tips with radial and flat geometric designs was evaluated. Among the protocols tested, the SWEEPS-R group showed the lowest residual medicament percentages and exhibited a higher removal efficiency compared to other activation methods. Similarly, Usta et al. [46] reported that SWEEPS activation was more effective than SNI and other activation techniques for removing CH and DAP in an immature tooth model. Korucu and Uğur Aydın [24] also investigated PIPS and SWEEPS tips with different geometric designs in an immature apex model and reported that radial tip designs may enhance hydrodynamic efficiency within the root canal. Although no previous study has directly evaluated the influence of fiber-tip geometry on intracanal medicament removal efficiency using PIPS and SWEEPS, experimental studies investigating fluid dynamics may help explain these observations. Gregorcic et al. [19] demonstrated that different Er:YAG laser fiber-tip geometries produce distinct cavitation patterns, with radial tips generating more spherical bubbles and flat tips producing channel-like bubble formations. Since the mechanical energy transferred to the irrigant is associated with bubble expansion dynamics, the more regular spherical bubbles produced by radial tips may facilitate greater energy transfer within the irrigant. However, it was also emphasized that an increase in the fiber tip diameter increases the bubble energy [19]. These hydrodynamic characteristics may contribute to the lower residual medicament values observed in the SWEEPS-R group in the present study.

This study compared the effectiveness of PIPS-F, PIPS-R, SWEEPS-F, and SWEEPS-R and demonstrated lower residual medicament values, particularly in protocols using the radial tip. Furthermore, the interaction between the irrigation activation method and medicament type was not statistically significant, indicating that the relative effectiveness of the tested irrigation activation techniques remained consistent regardless of whether CH or DAP was used. Despite these meaningful findings, the present study has limitations inherent to its in vitro design and therefore could not fully simulate clinical conditions. The evaluations were based on the percentage of medicament remaining on the root canal wall, and neither the possible penetration of the medicament into dentinal tubules nor its three-dimensional distribution within the root canal system was assessed using volumetric imaging methods such as micro-computed tomography. By comparing both medicament types and irrigation activation methods within the same experimental model, this study may help guide the selection of protocols for intracanal medicament removal in RET procedures. However, further well-designed clinical studies are needed to validate these findings.

## Conclusion

Within the limitations of this in vitro study, LAI significantly improved the removal of intracanal medicaments from an immature root canal model compared with conventional needle irrigation. Among the tested protocols, the SWEEPS mode applied with a radial tip demonstrated the lowest residual medicament values for both CH and DAP. However, none of the irrigation protocols achieved complete removal of the medicaments. Considering the potential biological effects of intracanal medicaments, including their ability to promote growth factor release from dentin, the optimal level of medicament removal in regenerative endodontic procedures should be interpreted cautiously and requires further clinical investigation.

## Data Availability

All data generated and analyzed during this study are included in the main text of the manuscript.
